# Transcranial Magneto-Acoustic Stimulation Protects Synaptic Rehabilitation from Amyloid-Beta Plaques via Regulation of Microglial Functions

**DOI:** 10.3390/ijms25094651

**Published:** 2024-04-24

**Authors:** Chunlan Zhang, Ruxin Tan, Xiaoqing Zhou, Ruru Wang, Xin Wang, Ren Ma, Fangxuan Chu, Ying Li, Tao Yin, Zhipeng Liu

**Affiliations:** 1 Institute of Biomedical Engineering, Chinese Academy of Medical Sciences & Peking Union Medical College, 236# Baidi Road, Tianjin 300192, China; yixuezcl@163.com (C.Z.); tanruxin78@hotmail.com (R.T.); xiaoqing-lingjun@163.com (X.Z.); wangruru_ruby@163.com (R.W.); wangxin121926@163.com (X.W.); maren@bme.cams.cn (R.M.); 2120181332@mail.nankai.edu.cn (F.C.); bme500@163.com (T.Y.); 2 Tianjin Institutes of Health Science, Tianjin 301600, China

**Keywords:** transcranial magneto-acoustic stimulation, transcranial ultrasound stimulation, neuromodulation, microglia, Alzheimer’s disease

## Abstract

Transcranial magneto-acoustic stimulation (TMAS), which is characterized by high spatiotemporal resolution and high penetrability, is a non-invasive neuromodulation technology based on the magnetic–acoustic coupling effect. To reveal the effects of TMAS treatment on amyloid-beta (Aβ) plaque and synaptic plasticity in Alzheimer’s disease, we conducted a comparative analysis of TMAS and transcranial ultrasound stimulation (TUS) based on acoustic effects in 5xFAD mice and BV2 microglia cells. We found that the TMAS-TUS treatment effectively reduced amyloid plaque loads and plaque-associated neurotoxicity. Additionally, TMAS-TUS treatment ameliorated impairments in long-term memory formation and long-term potentiation. Moreover, TMAS-TUS treatment stimulated microglial proliferation and migration while enhancing the phagocytosis and clearance of Aβ. In 5xFAD mice with induced microglial exhaustion, TMAS-TUS treatment-mediated Aβ plaque reduction, synaptic rehabilitation improvement, and the increase in phospho-AKT levels were diminished. Overall, our study highlights that stimulation of hippocampal microglia by TMAS treatment can induce anti-cognitive impairment effects via PI3K-AKT signaling, providing hope for the development of new strategies for an adjuvant therapy for Alzheimer’s disease.

## 1. Introduction

Alzheimer’s disease (AD) is a common neurodegenerative disease characterized by amyloid deposition [1,2]. The accumulation and impaired clearance of amyloid-β (Aβ) have been identified as key factors in the progression of AD [3]. Currently, strategies to enhance the clearance of Aβ still hold promise for AD treatment and prevention [4]. Microglia, the resident immune cells of the central nervous system, cluster around Aβ plaques and play a crucial role in Aβ clearance [5]. Recent studies have found that plaque-associated microglia enhance amyloid compaction and reduce plaque-associated toxicity, including dystrophic neurites, synaptic degradation, and cognition impairment [6,7]. However, as no approved treatment can revert or arrest the progression of AD, the development of new treatment methods for AD is urgently needed. Recent developments in TUS and TMAS have shown promise in modulating the microglial response to Aβ deposition.

Transcranial ultrasound stimulation (TUS) is a non-invasive neuromodulation technology based on acoustic effects. The research conducted by Bobola et al. demonstrated that TUS targeted the hippocampus of 5xFAD mice and reduced the Aβ plaque load, which was accompanied by microglial activation [8]. In addition, TUS covering the entire brains of 5xFAD mice reduced hippocampal Aβ plaques and improved microglial activation [9]. These findings suggest that TUS-mediated microglial activation will likely contribute to TUS-induced Aβ plaque reduction.

Transcranial magneto-acoustic coupling stimulation (TMAS) is a non-invasive neuromodulation technology that is based on the magnetic–acoustic coupling effect to generate a focused, coupled electric field in tissues. Previously, a study reported that TMAS was more effective than TUS in enhancing synaptic plasticity and improving memory ability in a mouse model of Parkinson’s disease (PD) [10,11]. Additionally, Yuexiang Wang et al. found that TMAS improved dendritic spine densities in the dentate gyrus region of PD mice [12]. Overall, the magnetic–acoustic coupling effect of TMAS is expected to produce a superior regulatory effect to TUS.

Alzheimer’s disease is characterized by lesions of the hippocampus, along with a dysfunctional memory process [13]. In the memory process of the hippocampus, the phosphatidylinositol-4,5-bisphosphate 3-kinase (PI3K) signaling axis has been shown to highly regulate both long-term potentiation (LTP) and long-term depotentiation (DEP) [14]. AKT (protein kinase B) is activated by upstream PI3K and is involved in hippocampus-dependent long-term memory consolidation [15]. Decreased levels of PI3K and p-AKT have been observed in the AD brain [16,17]. Accumulated studies have proven that the increased activation of PI3K and p-AKT enhances memory improvement [18,19,20]. The mechanisms underlying the synaptic plasticity and memory triggered by TMAS treatment are not yet clear. Here, we explore whether TMAS stimulates hippocampal microglia to promote Aβ clearance and cognitive improvement through activating the PI3K-AKT signaling pathway.

## 2. Results

### 2.1. The Schedule for TUS and TMAS Treatments for 5xFAD Transgenic Mice

**Figure 1:**
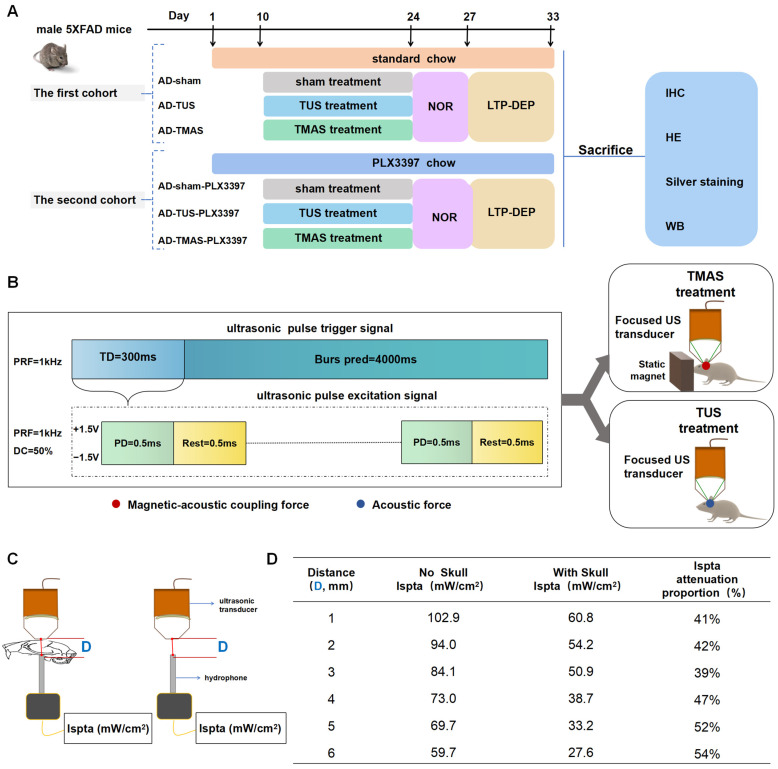
Flowchart of the experimental design for TMAS-TUS treatment. (**A**) Treatment plan for two cohorts of 5xFAD mice (NOR, novel object recognition test; LTP, long-term potentiation; DEP, depotentiation; IHC, immunohistochemistry; WB, Western blotting; HE, hematoxylin–eosin staining). (**B**) Ultrasonic parameters were set to a pulse trigger signal of 300 ms of excitation ultrasound. (**C**,**D**) The hydrophone detected ultrasonic energy (Ispta, mW/cm^2^); the farther away the skull surface, the more the energy attenuated.

TMAS-TUS treatment was applied for 14 days, starting on day 10 of oral PLX3397 administration. Following TMAS-TUS treatment, the novel object recognition (NOR) test was performed for 3 days. Subsequently, the LTP-DEP was recorded to evaluate synaptic plasticity in vivo for 6 days. After the mice were sacrificed, the histopathological investigation of mouse brain tissue and the potential signaling mechanism were examined (Figure 1A). The magnetic–acoustic coupling force from the TMAS experimental system was transmitted to the hippocampus through a focused ultrasound transducer and a static magnet. In contrast, the TUS experimental system only transmitted the acoustic force to the hippocampus. The ultrasound parameters of TUS and TMAS contained triggered ultrasounds, and a whole ultrasound signal held a 300 ms ultrasonic pulse excitation signal and a 4000 ms ultrasound interval (Figure 1B). We also measured the attenuation of energy when the ultrasound passed through the mouse’s skull, finding that the skull blocked 41% of the ultrasonic energy and the brain tissue further attenuated 13% of the ultrasonic energy (Figure 1C,D).

### 2.2. TMAS-TUS Treatment Promotes Activation of Microglia through the Cell Proliferation, Motility, and Phagocytic Capacities

Microglia, the primary immune cells in the central nervous system, play a crucial role in maintaining homeostasis. They constantly monitor and respond to both internal and external substances, exhibiting proliferation, migration, and phagocytic capacities [21,22]. Therefore, we employed IHC to examine the co-localization of CD11C and IBA1, indicating changes in the phagocytic state of the microglia. Furthermore, we utilized transwell and CCK-8 assays to investigate microglial cell migration and proliferation, respectively.

**Figure 2:**
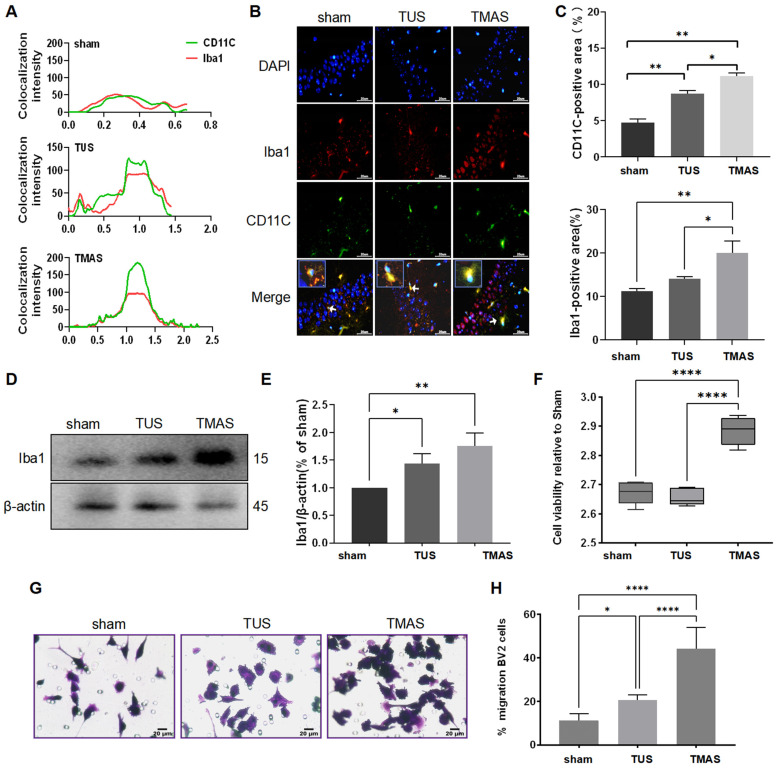
TUS and TMAS treatments induced proliferation, migration, and phagocytic capacities in microglial cells. (**A**) The co-localization analysis of CD11C (green) and Iba1 (red) using Image J 1.8.0. (**B**) The hippocampal sections of groups were stained with DAPI (blue) for nuclei, Iba1 (red) for microglia, or CD11C (green) for microglial phagocytosis (*n* = 3 mice, 6 fields from each group were used for analysis). Original magnification ×400; scale bar: 20 μm. (**C**) Quantitation of the CD11C and Iba1 fluorescence area within each slice. (**D**) Iba1 was analyzed with WB experiments after TUS and TMAS treatments of 5xFAD mice (*n* = 3 mice; we double checked each group for analysis) for two weeks. (**E**) Quantitative analysis of IBA1 level in D. (**F**) A CCK-8 assay detected the proliferation of BV2 cells after TMAS or TUS treatment. (**G**,**H**) Transwell analysis showed BV2 cell migration after TMAS or TUS treatment. All data in the results are expressed as mean ± SEM. * *p* < 0.05; ** *p* < 0.01; **** *p* < 0.0001; ns, not significant.

CD11C is an adhesion molecule that serves as a marker for microglial migration and phagocytosis [23,24]. The colocalization finder in Image J software 1.8.0 was used in the co-localization analysis of CD11C and Iba1. We drew a line from top to bottom in the particular area, and the co-location analysis was performed (Figure 2A). IHC analysis revealed that TMAS treatment significantly increased the expression levels of CD11C (TMAS vs. sham, *p* = 0.0016; TMAS vs. TUS, *p* = 0.0490) and Iba1 (TMAS vs. sham, *p* = 0.0011; TMAS vs. TUS, *p* = 0.0180) compared to sham- or TUS-treated mice (Figure 2B,C). Furthermore, the WB experiments also supported these findings, showing that TMAS or TUS treatment led to a higher protein level of Iba1 than the sham treatment group had (Figure 2D,E, TMAS vs. sham, *p* = 0.0039; TUS vs. sham, *p* = 0.0489). These results indicate that the microglia were more activated in the TMAS-treated group, which was additionally confirmed with the notable increase in the CD11C immunoreactivity area.

Blasi et al. developed BV2 cells, an immortalized mouse microglial cell line which has been widely used in studying brain immunity [25]. In our study, we investigated the effects of TMAS treatment and TUS treatment on the proliferation and migration of BV2 cells compared to a sham treatment. The results of a CCK-8 cell proliferation assay demonstrated that the results were higher in the TMAS treatment group than in the TUS and sham treatment groups (Figure 2F; TMAS vs. sham, *p* < 0.0001; TMAS vs. TUS, *p* < 0.0001). We observed that both TUS and TMAS treatments resulted in a higher number of transmigrated BV2 cells compared to the sham group, with the TMAS-treated group showing the highest number of cells (Figure 2G,H; TMAS vs. sham, *p* < 0.0001; TMAS vs. TUS, *p* < 0.0001; TUS vs. sham, *p* = 0.0310). These findings highlight TMAS as an effective regulator of BV2 cell function in all three treatment groups.

### 2.3. TMAS-TUS Treatment Enhances Microglial Phagocytosis and Clearance of Aβ

**Figure 3:**
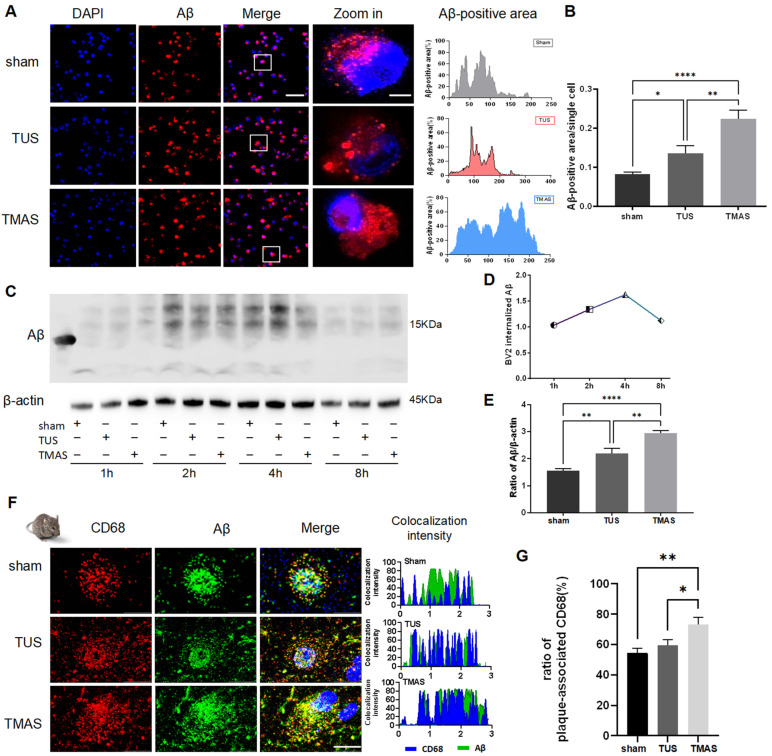
TMAS-TUS-treated microglia associated with Aβ plaque phagocytosis. (**A**,**B**) Confocal assays detected that BV2 cells engulfed 10 μM Aβ42 (HiLyte™ Fluor 488-labeled, human) after TMAS or TUS treatments. Original magnification ×20; scale bar: 100 μm. Zoom-in images on the right have a scale bar equal to 20 μm. (**C**–**E**) WB experiments showed the phagocytosis of Aβ by BV2 cells. (**F**) Coronal sections were stained with DAPI (blue) for nuclei, anti-Aβ (green) for Aβ plaques, or CD68 (red) for a phagocytic phenotype of microglia. Aβ colocalized with CD68 is shown in the last column of the figure. Original magnification ×40. Zoom-in images on the right have a scale bar equal to 20 μm. (**G**) Quantitation of the ratio of Aβ colocalized with CD68 within each plaque (*n* = 4 mice for sham group, *n* = 4 mice for TUS group, *n* = 3 mice for TMAS group; 20 Aβ plaques from sham group were used for analysis, 20 Aβ plaques from TUS group were used for analysis, 15 Aβ plaques from TMAS group were used for analysis). All data in the results are expressed as mean ± SEM. * *p* < 0.05; ** *p* < 0.01; **** *p* < 0.0001; ns, not significant.

Amyloid plaques play an important role in the pathogenesis of Alzheimer’s disease [26,27]. Microglial phagocytosis is essential for diminishing the accumulation of amyloid plaques [28,29]. In this study, we incubated BV2 cells with HiLyte™ Fluor-488-labeled Aβ and analyzed the fluorescence of BV2 endocytosis under a fluorescence confocal microscope (Figure 3A). The analysis measurement tool in Image J software 1.8.0 was used to analyze the fluorescence of Aβ in BV2 cells. The results showed that the amount of Aβ phagocytosed by BV2 cells in the TMAS-treated group was higher than in the TUS- (TMAS vs. TUS, *p* = 0.0055) and sham-treated groups (TMAS vs. sham, *p* = 0.0002) (Figure 3B). In addition, BV2 cells were cultured with 10 μM Aβ particles for 1 h, 2 h, 4 h, and 8 h after TMAS and TUS treatments. A WB experiment was performed to detect the levels of internalized Aβ in BV2 cells following the elimination of free Aβ from the cell culture medium by PBS (Figure 3C). Our results demonstrated that the peak of the BV2 cells’ phagocytosis of Aβ was at 4 h (Figure 3D), and TMAS treatment promoted BV2 cells to perform endocytosis of more Aβ than the sham group (TMAS vs. sham, *p* < 0.0001) and TUS group (TMAS vs. TUS, *p* = 0.0026) (Figure 3E).

For in vivo research, the colocalization finder in Image J 1.8.0 was used to perform quantitative analysis of the area of colocalization of Aβ with CD68, a phagocytic marker for microglia in the brain, further revealing phagocytic Aβ uptake by the microglia (Figure 3F). We found that the microglia in TMAS-treated 5xFAD mice contained more Aβ than what was observed in TUS- (TMAS vs. TUS, *p* = 0.0353) and sham-treated 5xFAD mice (TMAS vs. sham, *p* = 0.0089) (Figure 3G). Overall, the in vitro and in vivo evaluations indicate that TMAS likely activates Aβ phagocytic microglia.

### 2.4. TMAS-TUS Treatment Reduces Amyloid Burden and Related Toxicity

**Figure 4:**
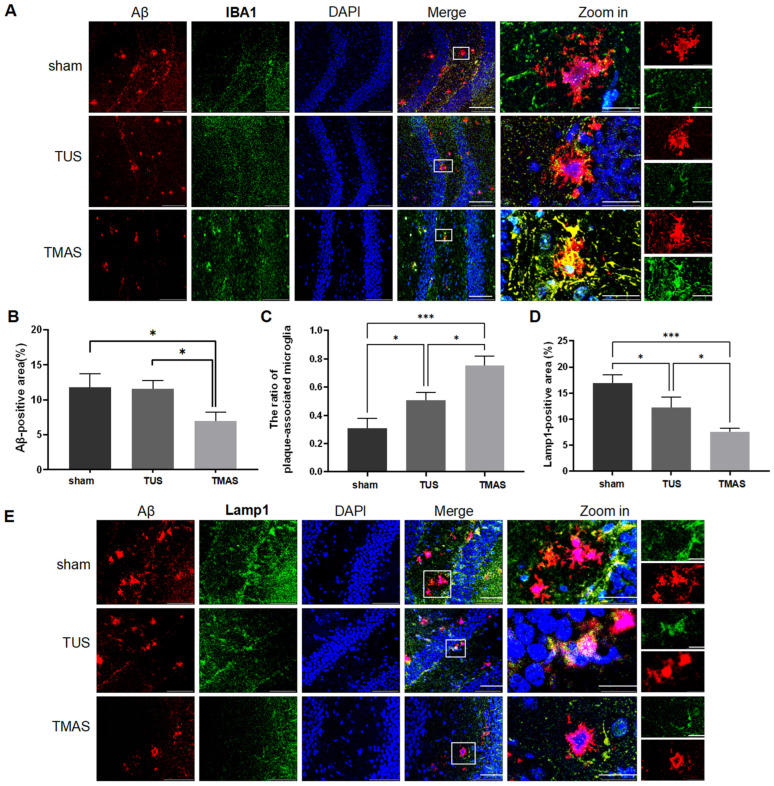
TUS and TMAS treatments attenuated amyloid burdens and amyloid-induced neurotoxicity. (**A**) Coronal sections of sham-, TUS-, or TMAS-treated 5xFAD mice were stained with DAPI (blue) for nuclei, anti-Aβ (red) for Aβ plaques, and Iba1 (green) for microglia. Representative images of the hippocampal regions are shown. Original magnification ×20; scale bar: 100 μm. (**B**) Statistical analysis of the difference in the Aβ fluorescence areas between sham, TUS, and TMAS groups (6 mice from each group were used for analysis). (**C**) Quantitation of the number of plaque-associated microglia in A (6 mice from each group were used for analysis). (**D**) Quantitation of the area of Lamp1-positive dystrophic neurites in E (6 mice from each group were used for analysis). (**E**) Coronal sections from sham-, TUS-, or TMAS-treated 5xFAD mice were stained with DAPI (blue) for nuclei, anti-Aβ (red) for Aβ, and Lamp1 (green) for dystrophic neurites. Representative images of the hippocampus regions are shown. Original magnification ×40; scale bar: 50 μm. Zoom-in images on the right have a scale bar equal to 20 μm. All data in the results are expressed as mean ± SEM. * *p* < 0.05; *** *p* < 0.001.

Alzheimer’s disease is characterized by abnormal amyloid deposition in the brain, leading to amyloid plaques [30]. This accumulation disrupts the microenvironment surrounding the plaques, causing a swelling of presynaptic terminals known as dystrophic neurites [31,32]. We tested whether TMAS-TUS-treated microglia could attenuate amyloid plaques and plaque-associated neurotoxicity in 5xFAD mice. After two weeks of TMAS-TUS treatment, the 5xFAD mice were sacrificed to analyze changes in the amyloid plaques. The brain tissue of 5xFAD mice was sectioned into coronal sections, which were then subjected to IHC analysis; we found that the TMAS treatment effectively decreased the amyloid plaque burden in comparison to the sham group (TMAS vs. sham, *p* = 0.0386) and TUS group (TMAS vs. TUS, *p* = 0.0475), as seen in Figure 4A,B.

In order to study the effects of TMAS-TUS treatment on microglial barrier function, we counted the plaque-associated microglia and found a more significant rise in the microglia population near the plaque following TMAS treatment compared to the TUS- and sham-treated groups, as shown in Figure 4C (TMAS vs. TUS, *p* = 0.0163; TMAS vs. sham, *p* = 0.0002). To analyze the dystrophic neurites, we used anti-Lamp1 as a marker for aberrant lysosomal accumulation in the dystrophic neurites around plaques [32] and found that TMAS treatment significantly reduced the overall area of the dystrophic neurites (TMAS vs. sham, *p* = 0.0006; TMAS vs. TUS, *p* = 0.0468; Figure 4D,E), which is consistent with its protective effects on amyloid deposition. In summary, TMAS treatment decreases amyloid deposition and enhances the microglial clustering around plaques, thus further attenuating the amyloid load and the neurotoxicity associated with plaques.

### 2.5. Microglia Are Necessary for TMAS-TUS Treatment-Induced Aβ Plaque Reduction

**Figure 5:**
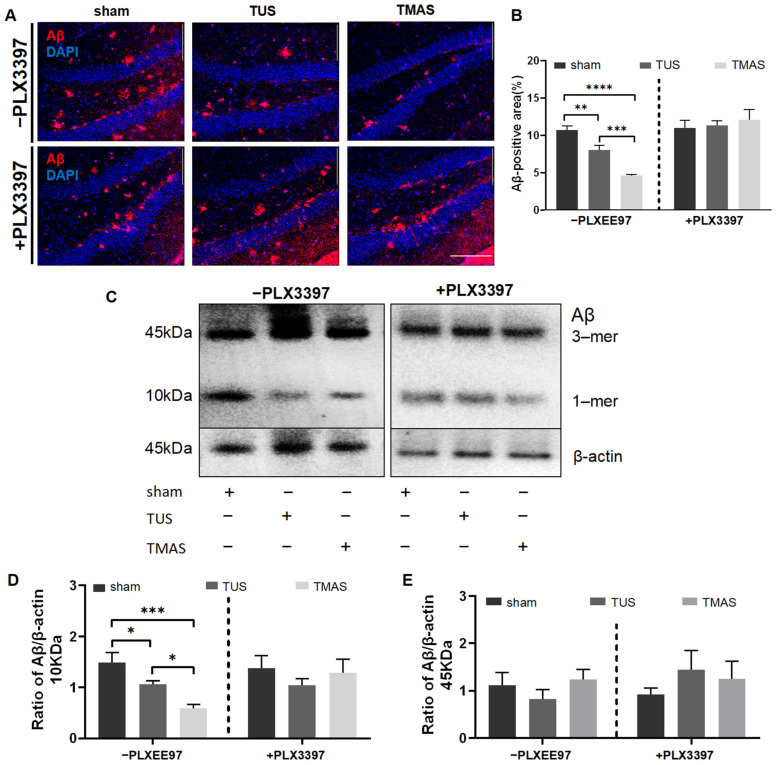
TUS and TMAS treatments no longer reduced amyloid plaques with administration of PLX3397. (**A**) Coronal sections were stained with anti-Aβ (red) for Aβ plaques and DAPI (blue) for nuclei. Original magnification ×20; scale bar: 100 μm. (**B**) Quantitation of the Aβ fluorescence areas in A (6 mice from each group were used for analysis). (**C**) WB experiments showed the difference between groups in 10 kD oligomeric Aβ and 45 kD trimeric Aβ. β-actin was used for normalization, as it is a major component of the cytoskeleton. (**D**) Quantitation of the 10 kDa Aβ expression in C (6 mice from each group were used for analysis). (**E**) Quantitation of the 45 kDa Aβ expression in C (6 mice from each group were used for analysis). All data in the results are expressed as mean ± SEM. * *p* < 0.05; ** *p* < 0.01; *** *p* < 0.001; **** *p* < 0.0001; ns, not significant.

To investigate whether microglia are responsible for the plaque reduction induced with TMAS-TUS treatment, we treated the 5xFAD mice with PLX3397 for 10 days, followed by continued administration of PLX3397 while subjecting the mice to 14 days of TMAS-TUS treatment. We found that the amyloid plaque burden was significantly decreased in the TMAS treatment group compared to the TUS and sham treatment groups (TMAS vs. sham, *p* < 0.0001; TMAS vs. TUS, *p* = 0.0002; Figure 5A,B). In contrast to the -PLX3397 group, the plaque load was no longer reduced by TUS treatment (TUS vs. sham, *p* = 0.8035) and TMAS treatment (TMAS vs. sham, *p* = 0.4626) in the +PLX3397 group (Figure 5B). To further analyze amyloid plaques, we detected both 10 kD oligomeric Aβ and 45 kD trimeric Aβ through WB experiments (Figure 5C); it was found that TMAS and TUS significantly reduced the expression of 10 kD Aβ (TMAS vs. sham, *p* = 0.0001; TUS vs. sham, *p* = 0.0313) in the -PLX3397 group, but the expression levels of 10 kDa Aβ were no longer reduced in the +PLX3397 group (F (2, 15) = 0.5955, *p* = 0.5638), as shown in Figure 5D. The expression levels of 45 kD Aβ were not significantly different between the -PLX3397 group (F (2, 15) = 0.8497, *p* = 0.4471) and the +PLX3397 group (F (2, 15) = 0.6441, *p* = 0.5391) (Figure 5E). Collectively, these findings indicate an important role for microglia in TMAS-TUS treatment-mediated Aβ plaque reduction.

### 2.6. Microglia Are Necessary for TMAS-TUS Treatment-Induced Synaptic Rehabilitation

#### 2.6.1. Effects of TMAS-TUS Treatment on NOR Tests

**Figure 6:**
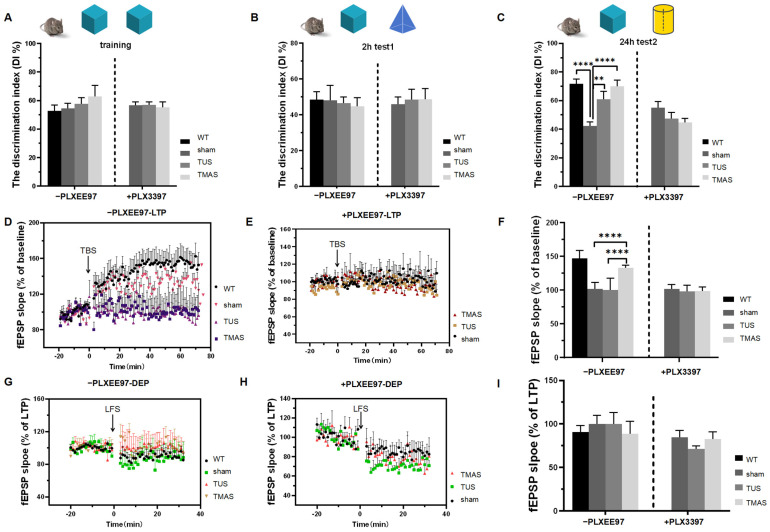
The NOR tests and electrophysiological recordings. (**A**) DI measurements of the -PLX3397 group and the +PLX3397 group during training phase (7 mice from each group were used for analysis). (**B**) DI measurements of the -PLX3397 group and the +PLX3397 group during Test 1 stage (7 mice from each group were used for analysis). (**C**) DI measurements of the -PLX3397 group and the +PLX3397 group during Test 2 stage (7 mice from each group were used for analysis). (**D**) The fEPSP of LTP of control chow-fed 5xFAD mice treated with TMAS-TUS treatment. (**E**) The averaged fEPSPs of LTP of PLX3397 chow-fed 5xFAD mice. (**F**) The averaged fEPSPs of LTP of (**D**,**E**) (6 mice from each group were used for analysis). (**G**) The fEPSP of DEP of control chow-fed 5xFAD mice. (**H**) The fEPSPs of DEP of PLX3397 chow-fed 5xFAD mice. (**I**) The averaged fEPSPs of DEP of (**G**,**H**) (6 mice from each group were used for analysis). All data in the results are expressed as mean ± SEM. ** *p* < 0.01; **** *p* < 0.0001; ns, not significant.

We assessed the learning and memory ability of 5xFAD mice after two weeks of TMAS-TUS treatment using the novel object recognition (NOR) test, which included a training phase, a short-memory testing stage (Test 1), and a long-term testing stage (Test 2) (Figure 6A–C). The discrimination index (DI) from the training phase showed no significant difference among the seven groups (F (6, 36) = 0.5253, *p* = 0.7853, Figure 6A), suggesting that all mice had comparable motor abilities. Test 1 showed no significant difference between all groups (F (6, 36) = 1.171, *p* = 0.3435, Figure 6B). However, in the Test 2 stage, the DI value of the TMAS-TUS-treated 5xFAD mice was significantly increased compared to the sham-treated 5xFAD mice (TMAS vs. sham, *p* < 0.0001; TUS vs. sham, *p* = 0.0035), and the sham-treated 5xFAD mice had a lower DI value than the WT group (sham vs. WT, *p* < 0.0001), as shown in Figure 6C. Then, we investigated whether microglia are required for the improvements in long-term memory formation induced through TMAS-TUS treatment and found that the improvement in long-term memory formation was diminished in the 5xFAD mice with induced microglial exhaustion in Test 2 (F (2, 18) = 1.898, *p* = 0.1787, Figure 6C). These results indicate that TMAS treatment improves the long-term memory formation, but not the short-term memory formation, of 5xFAD mice.

#### 2.6.2. Effects of TMAS-TUS Treatment on Electrophysiological Variation in the Hippocampus of 5xFAD Mice 

To determine the outcomes of TMAS-TUS treatment in synaptic plasticity-inducing long-term potentiation (LTP) and long-term depotentiation (DEP) in 5xFAD mice, next, we analyzed LTP and DEP after the NOR test. We found that TMAS treatment ameliorated the LTP impairment of 5xFAD mice as compared with the TUS group (TMAS vs. TUS, *p* < 0.0001) and the sham group (TMAS vs. sham, *p* < 0.0001), as shown in Figure 6D,F. However, there was no significant difference between groups in the DEP results (F (3, 220) = 2.409, *p* = 0.0680; Figure 6G,I). These findings indicate that TMAS treatment enhances memory formation. Then, we assessed if the microglia contributed to the LTP improvement induced with TMAS-TUS treatment and found that the LTP improvement was diminished in 5xFAD mice with induced microglial exhaustion (F (2, 150) = 1.092, *p* = 0.3383; Figure 6E,F). The DEP results showed no significant difference in the +PLX3397 group (F (2, 150) = 1.092, *p* = 0.3383; Figure 6H,I). Therefore, collectively, microglia play a crucial role in TMAS-TUS treatment-induced memory formation.

### 2.7. Effects of TMAS-TUS Treatment on Neuroplasticity-Associated Proteins and PI3K-AKT Signaling Protein Expressions in the Hippocampus of 5xFAD Mice 

**Figure 7:**
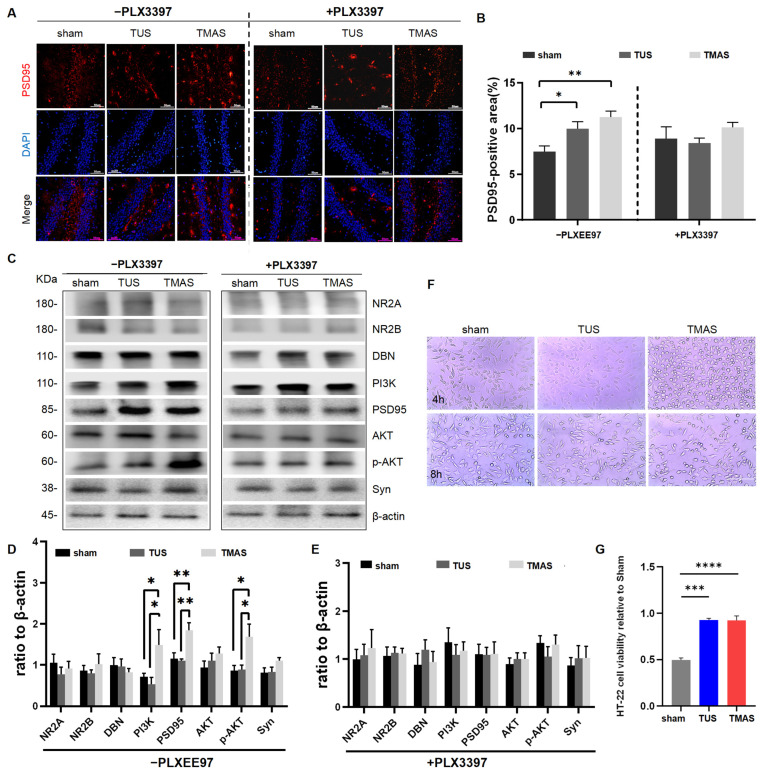
TMAS-TUS treatment-induced neuroplasticity-associated proteins and PI3K/AKT signaling protein expression. (**A**) Coronal sections were stained with DAPI (blue) for nuclei and PSD95 (red) for postsynaptic plasticity in the hippocampus. Representative images of the hippocampal regions are shown. Original magnification ×200; scale bar: 50 μm. (**B**) Quantitation of PSD95 fluorescence areas in A (*n* = 3 mice, 6 fields from each group were used for analysis). (**C**) Neuroplasticity-associated proteins and PI3K/AKT signaling protein expressions were analyzed with WB experiments. (**D**) Quantitative analysis of protein expression levels in the -PLX3397 group (6 mice from each group were used for analysis). (**E**) Quantitative analysis of protein expression levels in the +PLX3397 group (6 mice from each group were used for analysis). (**F**) Supernatants from a 24 h BV2 cell culture were collected after TUS and TMAS treatments and used to culture HT-22 cells for 24 h. Original magnification ×20; scale bar: 100 μm. (**G**) Quantitative analysis of the proliferation of HT-22 cells in F. All data in the results are expressed as mean ± SEM. * *p* < 0.05; ** *p* < 0.01; *** *p* < 0.001; **** *p* < 0. 0001; ns, not significant.

To further investigate the enhanced LTP effect in the hippocampus upon TMAS-TUS treatment, we measured the levels of neuroplasticity-related proteins, such as synaptophysin (Syn) and postsynaptic density 95 (PSD95), which are located at the presynaptic terminals and the postsynaptic density of neurons, respectively. According to our IHC results, the positive area of PSD95 was significantly increased with the TMAS-TUS treatment compared with sham-treated 5xFAD mice (TMAS vs. sham, *p* = 0.0078; TUS vs. sham, *p* = 0.0409); however, the TMAS-TUS treatment had no significant effect on the positive area of PSD95 in the absence of microglia (F (2, 6) = 1.095, *p* = 0.3933), as shown in Figure 7A,B. Furthermore, our WB results showed that TMAS treatment significantly improved the expression levels of PSD95 in 5xFAD mice (TMAS vs. sham, *p* = 0.0026; TMAS vs. TUS, *p* = 0.0015; Figure 7C,D) and also no longer altered the expression level of PSD95 in the +PLX3397 group (F (2, 15) = 0.001875, *p* = 0.9981; Figure 7C,E). Syn, as another prominent neuroplasticity-related protein, did not exhibit a significant group factor between the +PLX3397 group (F (2, 15) = 0.1512, *p* = 0.8610) and the -PLX3397 group (F (2, 15) = 2.387, *p* = 0.1258) (Figure 7D,E). The expression levels of other proteins associated with neuroplasticity included the N-methyl-D-aspartate (NMDA) receptor subunits 2A (NR2A) and 2B (NR2B), as well as drebrin (DBN), a functional protein in dendritic spines, and no significant differences were demonstrated in their levels in the -PLX3397 group (NR2A: F (2, 15) = 0.5625, *p* = 0.5813; NR2B: F (2, 15) = 0.4902, *p* = 0.6220; DBN: F (2, 15) = 0.3337, *p* = 0.7214) or the +PLX3397 group (NR2A: F (2, 15) = 0.1694, *p* = 0.8458; NR2B: F (2, 15) = 0.06236, *p* = 0.9398; DBN: F (2, 15) = 0.5791, *p* = 0.5724), as seen in Figure 7D,E.

To better understand the potential signaling mechanism following TMAS treatment, we examined the levels of various proteins involved in PI3K-AKT signaling. We found that TMAS treatment improved the expression levels of PI3K and p-AKT to a more significant degree than was found in the sham- and TUS-treated groups (PI3K: TMAS vs. sham, *p* = 0.0410; TMAS vs. TUS, *p* = 0.0143) (p-AKT: TMAS vs. sham, *p* = 0.0104; TMAS vs. TUS, *p* = 0.0124). However, the TUS and TMAS treatments could no longer increase the expression levels of PI3K and p-AKT in the absence of microglia (PI3K: F (2, 15) = 0.3239, *p* = 0.7283) (p-AKT: F (2, 15) = 0.7085, *p* = 0.5082). The expression levels of AKT showed no significant differences between the -PLX3397 group (F (2, 15) = 1.086, *p* = 0.3626) and the +PLX3397 group (F (2, 15) = 0.2095, *p* = 0.8133). These results are shown in Figure 7D,E.

Then, we also discovered that after TUS and TMAS treatments, HT-22 cells incubated with BV2 cell supernatant significantly proliferated in the TMAS and TUS treatment groups as compared with sham treatment group (TMAS vs. sham, *p* < 0.0001; TUS vs. sham, *p* = 0.0001; Figure 7F,G).

In summary, TMAS treatment exerted anti-cognitive impairment effects in a microglia-dependent manner via promoting PI3K-AKT signaling in the hippocampus.

## 3. Discussion

TMAS is widely acknowledged as a non-invasive neuromodulation technology; however, its impact on amyloid deposition and microglial functions in Alzheimer’s disease remains elusive. We conducted a comparison study of TMAS and TUS and observed that the TMAS-TUS treatment improved microglial functions, including proliferation, migration, and Aβ phagocytosis and clearance. Additionally, TMAS-TUS treatment also reduced amyloid deposition, alleviated recognition memory deficits, and improved LTP-like hippocampal synaptic excitations. Notably, microglial exhaustion lessened the TMAS-TUS-mediated Aβ plaque reduction and the enhancement of synaptic rehabilitation, highlighting that microglia play an important role in TMAS-TUS treatment-induced protective effects. Our research reveals a crucial link between the response of the microglia and the benefits of TMAS treatment.

Recent research has indicated that TUS monotherapy can enhance synaptic rehabilitation at 250–720 I_SPTA_ (mW/cm^2^) [33,34,35,36]; the I_SPTA_ used was 5 to 14 times higher than the 53 mW/cm^2^ we utilized. Additionally, the combination of ultrasound with microbubbles to invasively open the blood–brain barrier also enhances synaptic rehabilitation [37,38,39]. Studies on the transcranial static magnetic field, with strengths ranging from 0.69 T to 2 T+, have shown that it exerts a neuroregulatory effect. These magnetic field strengths are higher than our static magnetic field strength 0.15 T. In our TMAS system, TUS [10,11] and the static magnetic field were sub-threshold stimulations [40,41]. Our findings revealed that TMAS enhanced memory formation and synaptic rehabilitation via PI3K-AKT signal activation when compared to sub-threshold TUS. Furthermore, the improvements in TMAS performance can be attributed to the combined effects of the sub-threshold static magnetic field and the sub-threshold ultrasound field. Thus, future research is imperative for gaining a deeper understanding of the TMAS mechanism, along with elucidating the disparities in the functional mechanisms between the static magnetic field and the ultrasound field.

Transcranial ultrasound stimulation reduced amyloid deposition and activated the microglia [8,9], indicating a potential interaction between microglial activation and TUS-induced Aβ plaque reduction. Moreover, TMAS improved the cortical motor function and memory ability better than TUS [11], inducing speculation that the magnetic–acoustic coupling effect of TMAS may help generate a better regulatory effect than TUS. Hence, it is probable that TMAS acts as a stronger physical attractant for microglial recruitment to the location of a plaque than TUS. In fact, we found that TMAS was more effective in improving microglial functions and enhancing synaptic rehabilitation than TUS. Remarkably, PLX3397, a selective CSF1R kinase inhibitor that eradicated microglia from the 5xFAD mice, abolished the substantial effects of TMAS and TUS on amyloid deposition and synaptic rehabilitation through microglial depletion. Therefore, our data support that these significant differences are related to the microglial activation induced via the TMAS and TUS treatments. However, the microglial surface channel proteins that represent the substantial effects of TMAS and TUS remain unknown; therefore, further studies are required in this area. Microglial channel proteins include voltage-gated ion channels (potassium [42], sodium [43], calcium [44], and chloride channels [45]) and ultrasound-sensitive channel proteins (mechanosensitive (MSC) [46], Piezo [47], and transient receptor potential (TRP) [48]). Although our study has demonstrated that TMAS and TUS reverse cognitive and synaptic deficits depending on the microglia, the microglial neurotrophic functions of TMAS and the substantial TUS-induced effects on synaptic plasticity remain unknown. Previous research has indicated that microglia promote learning-dependent synapse formation through brain-derived neurotrophic factor [49], insulin-like growth factor [50], and cytokines (IL-33 [51], TNF [52], and IL-1β [53]). Therefore, TMAS may have a more effective influence than TUS on synaptic plasticity through boosting the neurotrophic functions of microglia.

In previous studies, a static magnetic field (SMF) significantly increased skin microcirculatory blood flow [54] and exerted certain influences on the diameter of fine veins and arteries, as well as blood flow velocity in the microcirculation of mouse auricles [55]. Additionally, an SMF enhanced arterial baroreflex sensitivity [56]. Exposure to an SMF led to a decrease in plasma NO(x) concentrations, along with reduced levels of angiotensin II and aldosterone [57]. Based on these results, this indicates that SMFs play a significant role in the modulation of blood metabolism, possibly through the nitric oxide (NO) signaling pathway and via hormonal regulatory mechanisms. Furthermore, SMFs exert an influence on neuronal activity, encompassing the liberation of neurotransmitters, the activation of ion channels situated on the neuronal cell membrane, and alterations in the threshold of action potential. The research has demonstrated that SMFs elevate the overall serotonin concentration. Nevertheless, its impact on the other three neurotransmitters, such as choline, γ-aminobutyric acid (GABA), and dopamine, remains minimal [58]. Furthermore, recordings of GlyH-101-sensitive Cl-current components using whole-cell voltage-clamp techniques have demonstrated a substantial increase in both sub-threshold and depolarized membrane potentials upon the application of an SMF [59]. In the future, it is imperative to persist in the examination of the underlying neural mechanisms of TMAS, which possess the potential to fortify cerebral blood flow, stimulate the ion channels residing on neuronal cell membranes, modulate the threshold of neuronal action potential, and regulate the discharge of neurotransmitters.

Magnetic–acoustic nanoparticles are targeted using magnetic fields, and drug delivery is facilitated through the application of ultrasounds [60,61,62]. It is important to note that the static magnetic field and ultrasounds are utilized independently, without any overlap or integration. However, the TMAS leverages the combined powers of a static magnetic field and an ultrasound to achieve a magneto-acoustic coupling effect. Classical piezoelectric materials that have demonstrated piezocatalytic properties include inorganic compounds such as BaTiO3, ZnO, and BiFeO3, alongside organic polyvinylidene fluoride (PVDF) [63,64]. These materials are notable for their high dielectric constants, excellent ferroelectric properties, and acceptable biocompatibility, making them widely exploited in clinical practice for treating a range of pathological conditions [65,66]. Furthermore, piezoelectric materials have been combined with Fe_2_O_4_, such as core–shell-structured CoFe_2_O_4_-BiFeO_3_ magnetostrictive–piezoelectric nanoparticles [67]. This combination allows TMAS to accurately transmit the magneto-acoustic coupling effect through materials similar to CoFe_2_O_4_-BiFeO_3_ nanoparticles. In addition, simultaneous multi-target ultrasound neuromodulation based on a single-element ultrasound transducer facilitates concurrent neuron stimulation across multiple brain areas and enables the exploration of novel neural circuits [68,69]. In contrast to optogenetics [70], which necessitates the introduction of exogenous gene expression to modify the inherent characteristics of the brain, the future holds the promise of precise neural regulation achieved through the integration of TMAS with magnetic–acoustic nanoparticles. This approach offers the advantage of neural regulation without altering gene expression, and thereby favoring its utilization in neural regulation research and medical applications. Given that our TMAS system incorporates both low-intensity ultrasound and low-strength static magnetic fields, it is capable of ensuring the safety of its prolonged, uninterrupted use in the context of chronic conditions such as Alzheimer’s disease, Parkinson’s disease, depression, and movement disorders.

The application of brain stimulation should be carried out within safety boundaries. We considered many aspects of ultrasonic parameters, including the ultrasonic density, exposure time, fundamental frequencies, and ultrasonic heat generation. To avoid long-term exposure and potentially excessive high intensities of ultrasound during brain modulation, it was necessary to limit the ultrasonic intensity and exposure time. In our study, we adopted a 53 mW/cm^2^ I_SPTA_ ultrasonic intensity, which was not only below the maximum 94 mW/cm^2^ limit for diagnostic imaging applications of fetuses and neonates but also below the 180 mW/cm^2^ limit for diagnostic imaging applications of adult human brains [71,72]. In addition, the ultrasonic intensity in our study was far lower than that used to regulate the activity of the primary somatosensory cortex in the human body, which was 23.87 W/cm^2^ [73]. In addition to the limitations on the maximum ultrasonic intensity, we also needed to ensure that the heat generated via a complete ultrasonic process did not exceed the normal physiological temperature (37 °C) by 1.5 °C [10]. Duck et al. found that higher temperature elevations from diagnostic exposure were limited to 41 °C (embryonic and fetal in situ temperatures) for 5 min, with anything beyond this considered potentially hazardous [74]. In the future, we should further detect the degree of warming of TMAS or TUS in the mouse brain. Prior studies have confirmed that low-intensity focused ultrasound pulsation (LIFUP), with a pulse repetition rate of 10 to 1000 Hz, can effectively regulate the activity of neurons [75,76]. Moreover, a 650 kHz ultrasound reduced energy loss when the ultrasound passed through the skull [77]. In addition, fundamental frequencies from 200 to 700 kHz did not affect the neuronal firing pattern. Based on these previous studies, we established a PRF of 1000 Hz and a duty factor of 50% in combination with a 500 kHz fundamental frequency and a single exposure of 5 min. In order to avoid the potential excessive immune activation associated with ultrasonic stimulation in the brain, the ultrasound may need to be applied with a lower intensity and smaller focus site. Recent advances in neuromodulation technology, such as temporal–spatial interference magneto-acoustic stimulation, possibly make a magneto-acoustic coupling current occur at the ultrasonic focus site. The 5xFAD transgenic AD mouse model only represents amyloid-related pathological processes and does not reflect the pathological processes of tau. Hence, the potential impact of TUS and TMAS on tau neurofibrillary degeneration remains to be investigated. Additionally, we only utilized male mice; therefore, in future studies, age-matched female mice will be important in investigating the effects of TMAS and TUS treatments on the pathological processes of AD.

In summary, our results demonstrate that TMAS enhances microglial functions to reduce amyloid plaque deposition and enhance synaptic rehabilitation, indicating that the effects of TMAS are superior to those of TUS in a microglial-dependent manner via the PI3K-AKT signaling pathway, providing new insights into the potential of TMAS for the treatment of Alzheimer’s disease.

## 4. Materials and Methods

### 4.1. Study Design

This study aimed to investigate how TUS and TMAS treatments affected the Aβ plaque load and anti-cognitive impairment of 5xFAD mice. Therefore, we treated and divided the 5xFAD mice into two cohorts. The first cohort of hemizygous male 5xFAD mice (median age, 6 months) was randomly assigned to the TUS, TMAS, or sham group (*n* = 7 per group) and received a 5-min daily treatment for 2 weeks (Figure 1D); this included a group of 7 wild-type mice. Then, we measured the effects of TUS and TMAS treatments on the amyloid pathology and synaptic plasticity in the hippocampus. A second cohort of hemizygous male 5xFAD mice (median age, 6 months) received microglial ablation using the microglia inhibitor PLX3397 (Topbiochem, Ltd., Shanghai, China; 1029044-16-3, 290 mg/kg formulated in standard chow). Subsequently, the microglia-deficient 5xFAD mice were randomly assigned to the TUS, TMAS, or sham group (*n* = 7 per group) and received a 5-min daily treatment for 2 weeks; this included a group of 7 wild-type mice. Then, we verified the effects of the TUS and TMAS treatments on amyloid pathology and synaptic plasticity in this second cohort. The treatment conditions were blinded until the analyses of the pathological results of all animals were completed.

### 4.2. Animals

The experiments were performed on 6-month-old male 5xFAD transgenic mice. Animals weighing 25–30 g were obtained from the Beijing HFK bioscience company. A 12 h light/dark cycle was used, with the lights switched on at 7:00 a.m. The first cohort of 5xFAD mice were fed standard chow. The second cohort of 5xFAD mice were fed the microglial inhibitor PLX3397 mixed in the chow. Water was available ad libitum. All animal experimental procedures met the experimental requirements of the Institute of Radiation Medicine, Chinese Academy of Medical Sciences and Peking Union Medical College.

In general, 5xFAD transgenic mice carry two major types of mutations. The first type is the precursor protein 695 (APP) mutation, including the Swedish (K670N and M671L), Florida (I716V), and London (V717I) mutations. The second type is the human Presenilin-1 (PS1) mutation carried by familial Alzheimer’s disease (FAD), including M146L and L286V. These mice almost exclusively and rapidly accumulate Aβ in the brain. Therefore, 5xFAD mice have amyloid plaques that are characteristic of AD.

### 4.3. TMAS-TUS Experimental System and Treatment

The ultrasonic signal generation was divided into two parts. The first part was the ultrasonic-induced signal (TFG6920A; Shu Ying, Shenzhen, China); the second part was the ultrasonic excitation signal (AFG3252; Tektronix, Beaverton, OR, USA); the two parts then joined a radio frequency amplifier (GA2500; Teclab Limited, Milwaukee, WI, USA) to form the completed ultrasonic signal. The ultrasonic signal was delivered to the subject via a focused US transducer (FP-0.5M; IOA-AC, Beijing, China) with a central frequency of 500 kHz. A needle hydrophone (developed by the Institute of Acoustics, Chinese Academy of Sciences Beijing) detected the sound pressure with a sensitivity of 2 µV/Pa. The sonication parameters of TUS were as follows: 0.6 ms TBD (tone-burst duration), 1 kHz PRF (pulse repetition frequency), 400 ms SD (sonication duration), and 106 mW/cm^2^ I_sppa_. Unlike the TUS equipment, the TMAS equipment had a permanent magnet. The static magnetic field strength of the permanent magnet was measured with a Gauss meter (model 475; Lakeshore, Columbus, OH, USA), and the magnetic field strength at the hippocampus of the mice was 0.15 T. The TMAS experimental system for animals (TMAS-ESA-BME01) was designed at the Institute of Biomedical Engineering, Chinese Academy of Medical Sciences and Peking Union Medical College. The TMAS-TUS experimental system (TMAS-ESA-BME01) involved the use of two function signal generators to produce the ultrasonic stimulation signal. The transducer, which converted the ultrasonic signal, was securely mounted onto the stereotactic locator’s three-axis bracket (SR-6M; Chengmao, Tokyo, Japan), enabling precise targeting through adjustments along the three axes. Simultaneously, a static magnet was positioned perpendicularly to the propagation direction of the ultrasonic wave of the transducer. The specification of the transducer and the distribution of the ultrasound pressure field were shown in Figure S1.

During the TUS and TMAS treatments, mice were anesthetized with isoflurane (RWD; Cat# R510-22-10, China, Shenzhen, China) in the breathing anesthesia machine (R580S; RWD, Shenzhen, China), and each mouse’s head was fixed on a stereotaxic frame with its head hair shaved. The ultrasonic probe was centered at 3.5 mm to the posterior of the bregma, along the midline, 1.9 mm below the skin surface, and covering the bilateral hippocampus. The focused ultrasound region was 4 mm in diameter. In the sham group, mice were stimulated by keeping the ultrasound probe and the static magnetic field turned off. All the mice received TMAS, TUS, or sham stimulation once per day for 2 weeks.

### 4.4. Silver Staining

After each mouse was sacrificed, the mouse brain was processed for silver staining [78,79]. Paraffin sections were dewaxed and rehydrated, treated using a silver glycine staining kit (Servicebio, Wuhan, China, Cat# G1052-500T), covered with silver glycine staining solution C for 5 min, treated with silver glycine staining solution B for 5 min, and then treated with silver glycine staining solution A for light staining. Sections were dehydrated three times with absolute ethanol and neutral resin. Stained sections were scanned under a white-field microscope and analyzed using Image J software 1.8.0.

### 4.5. Cell Culture and Proliferation Assay

The BV2 and HT-22 cell lines were purchased from the China Center for Type Culture Collection. The cells were cultured in Dulbecco’s modified Eagle medium supplemented with 1 × GlutaMAX^TM^-I, 5% (*v*/*v*) fetal bovine serum, 50 U/mL of penicillin, and 50 µg/mL of streptomycin, and cells were maintained at 37 °C and 5% CO_2_. Then, cell proliferation was detected with a CCK-8 kit (MedChemExpress, Shanghai, China; Cat# HY-K0301), as previously described in [80].

### 4.6. Aβ Phagocytosis Assays of BV2 Cells

BV2 cells at a density of 2 × 10^6^ cells were inoculated into a 60 mm culture dish. After TUS, TMAS, or sham treatment, cells were treated with the phagocytosis inhibitor cytochalasin D (Calbiochem, Saint Louis, MO, USA; Cat#250255, RRID:HY-19693/cs-6968, 10 µM) for 30 min [81], and incubation using HiLyte™ Fluor 555-labeled-Aβ42 oligomers (Anaspec, Fremont, CA, USA; Cat# AS-60480-01, 500 nM) for an additional 4 h followed [82]. Cold PBS-washed cells were used for the detection of Fluor 555-labeled Aβ42 fluorescence under a confocal microscope.

### 4.7. Microglial Migration in Transwell Assays

BV2 cells suspended in 100 μL of serum-free DMEM medium were inoculated into the upper cavity of the transwell. A total of 600 μL of serum-free DMEM medium was added into the lower cavity of the transwell. Subsequently, the transwell was incubated in a cell incubator (5% CO_2_) for 1 h. Next, the serum-free DMEM medium in the lower cavity of the transwell was replaced with 10 μM of Aβ42 (Macklin, Shanghai, China; Cat# 107761-42-2) serum-free DMEM medium [83,84]. The transwell continued to be incubated in the incubator for 24 h. The BV2 cells on the membrane surface were removed with cotton swabs and washed with PBS three times. Next, the cells were fixed with 4% polyformaldehyde (PFA) for 20 min. After the cells were stained with crystal violet staining solution, they were photographed and recorded under a white-field microscope.

### 4.8. Western Blotting

Mice were anesthetized and 50 mL of pre-chilled 1× PBS was perfused through the hearts. The brains were dissected and divided at the midline. One side of the brain was further dissected, and the hippocampus was quickly frozen in liquid nitrogen and stored at −80 °C. Hippocampal tissue was immersed in RIPA lysis and extraction buffer (Beyotime; Cat# P0013B, Shanghai, China) supplemented with a protease and phosphatase inhibitor cocktail (Beyotime; Cat# P1051, Shanghai, China). The total protein was precipitated through centrifugation, and the protein concentration was determined with a BCA protein assay kit (Beyotime; Cat# P0009, Shanghai, China). Equal amounts of total protein from each group were obtained using Sure PAGE™ gels (GenScript; Cat# M00654, NJ, USA) and transferred to polyvinylidene fluoride (PVDF) membranes (Solarbio; Cat# ISEQ00010, Beijing, China). The PVDF membranes were blocked and then incubated with primary antibodies overnight at 4 °C. After washing, the PVDF membranes were incubated with the secondary antibody for 1 h, and enhanced chemiluminescence Western blotting detection reagents (Solarbio; Cat# PE0010, Beijing, China) were used after washing to visualize the bands. The grayscale values of each band were measured using Image J 1.8.0 and then compared with the grayscale values of the corresponding β-actin band. This comparison was performed through the calculation of the relative grayscale values or by calculating ratios. The following antibodies were used: anti-Iba1 (Wako; Cat# 016-20001, RRID: AB_839506, 1:500, Osaka, Japan), anti-Aβ_1–42_ (Cell Signaling Technology; Cat# D9A3A, RRID: AB_2798671, 1:1000, Danvers, MA, USA), anti-PSD95 (Cell Signaling Technology; Cat# 3450S, RRID: AB_2292883, 1:2000, Danvers, MA, USA), anti-synaptophysin (Sigma-Aldrich; Cat# S5768, RRID: AB_477523, 1:2000, Saint Louis, MO, USA), anti-NR2A (Millipore; Cat# 07-632, RRID: AB_310837, 1:1000, Danvers, MA, USA), anti-NR2B (Cell Signaling Technology; Cat# 14544, RRID: AB_2798506, 1:1000, Danvers, MA, USA), anti-DBN (ThermoFisher; Cat# MA5-28543, RRID: AB_2745502, 1:1000, Waltham, MA, USA), anti-PI3K (Cell Signaling Technology; Cat# 3821, RRID: AB_330320, 1:1000, Danvers, MA, USA), anti-AKT (Abcam; Cat# ab8932, RRID: AB_306867, 1/500, Cambridge, UK), anti-p-AKT (Abcam; Cat# ab8805, RRID: AB_306791, 1/500, Cambridge, UK), and anti-β-actin (Cell Signaling Technology; Cat# 12262, RRID: AB_2566811, 1:5000, Danvers, MA, USA).

### 4.9. Immunohistochemistry and Microscopy

The other halves of the mouse brains were fixed in 4% paraformaldehyde (Invitrogen; Cat# J61899, Shanghai, China) overnight at 4 °C; then, the brains were dehydrated and paraffin-embedded, followed by sectioning. The paraffin sections were dewaxed with xylene and absolute ethanol; then, the sections were placed in an antigen retrieval buffer (pH 6.0) with citric acid (Solarbio; Cat# C1032, Beijing, China) for antigen retrieval. The sections were blocked with a Triton X-100 permeabilization solution (BIOSS, Cat# P0096, Beijing, China) and a 1% bovine serum albumin-blocking solution (BIOSS; Cat# bs-0292P, Beijing, China). Then, the sections were incubated in a primary antibody overnight at 4 °C in a humidified chamber. The sections were covered with a fluorophore-containing secondary antibody (CY3) and incubated for 50 min at room temperature in the dark. The nuclei were counterstained with a DAPI staining solution (BIOSS; Cat# C02-04002, Beijing, China) added dropwise onto the sections and incubated for 10 min at room temperature in the dark. After drying, the sections were mounted with an anti-fluorescence quenching mounting medium (Solarbio; Cat# S2110, Beijing, China) and stored in the dark at 4 °C after mounting. Each section in Figure 2B, Figure 7A and Figure S2 was observed under a fluorescence microscope and photographed at three magnifications: 100×, 200×, and 400×. Each section in Figure 3A,F, Figure 4A,E and Figure 5A was observed under a fluorescence confocal microscope and photographed at two magnifications: 20× and 40×. For analysis, the selected file was initially input into the Image J software 1.8.0, and then the colocalization finder module was used to analyze the colocalization of multiple fluorescence signals. The colocalized area was calculated in the adjust threshold module, and the proportion of fluorescence occupied by colocalization was determined through comparison of the colocalized area with the fluorescence area in a specific channel. The primary antibodies used were as follows: anti-Iba1 (Wako; Cat# 016-20001, RRID: AB_839506, 1:500, Osaka, Japan), anti-Iba1 (Santa Cruz Biotechnology; Cat# sc-32725, RRID: AB_667733, 1:500, Santa Cruz, CA, USA), anti-Aβ (Abcam; Cat# ab32136, RRID: AB_2289606, 1/500, Cambridge, UK), anti-PSD95 (Cell Signaling Technology; Cat# 3450S, RRID: AB_2292883, 1:2000, Danvers, MA, USA), anti-CD11c (Biolegend; Cat# 117304, RRID: AB_313773, 1:1000, Santa Cruz, CA, USA), anti-Lamp1 (BIOSS; Cat# bs-1970R, 1:100, Beijing, China), and anti-CD68 (Bio-Rad; Cat# MCA1957, RRID: AB_322219, 1:50, Heraklion, CA, USA). Appropriate secondary antibodies were used from the Alexa Series from Molecular Probes.

### 4.10. Novel Object Recognition (NOR) Test

The NOR test assessed learning and memory in mice after two weeks of TMAS-TUS treatment. This test involved an opaque box (50 × 50 × 36 cm) with a camera recording the session. The NOR test consisted of four stages: adaptation, training, a short-term memory test, and a long-term memory test. In the adaptation stage, mice were familiarized with the box without any objects. During the training stage, mice were trained with two identical objects (object A1 and object A2) for 10 min. At 2 h after the training stage, in the short-memory testing stage (Test 1), object A2 was replaced with a new object, B. Mice were then allowed to freely explore the objects (object A1 and object B) for 10 min. Then, 24 h after the training stage, in the long-term testing stage (Test 2), object B was replaced with a new object, C, and mice were again given 10 min to freely explore the objects (object A1 and object C). An imaging system recorded the time mice spent on novel and old objects. Hence, we defined the discrimination index (DI = novel object exploration time/total exploration time × 100%) to assess the learning and memory capacity of each mouse. The box and objects were sanitized with 70% ethanol following each trial; it was ensured that the sanitization process was meticulous to eliminate any odors.

### 4.11. In Vivo Electrophysiological Study

After the NOR test, both the 5xFAD mice and the wild-type mice underwent electrophysiological experiments. The long-term potentiation (LTP) and long-term depotentiation (DEP) protocols were similar to those in prior studies [85,86]. During the electrophysiological experiments, the mice were anesthetized with 30% urethane and placed on a stereotaxic frame. A scalp incision was made to expose the skull, and a hole was drilled for the insertion of the recording and stimulating electrodes. A bipolar stimulating electrode was implanted in the PP (−3.8 mm AP, 3.0 mm ML, and 1.5 mm DV) of the hippocampus. A recording electrode was positioned in the DG (−2.0 mm AP, 1.4 mm ML, and 1.5 mm DV) of the hippocampus. The positions of the electrodes were optimized through adjustment of their depths while recording the response to a single stimulation pulse with the parameters of 0.2 ms duration and 0.4 mA intensity. An input–output (I-O) curve was then obtained through varying the stimulation intensity from 0.1 to 1 mA in increments of 0.1 mA. The stimulation intensity that evoked a 50–60% field excitatory postsynaptic potential (fEPSP) was selected for the subsequent experiments. The basal fEPSP was recorded every 60 s for 30 min. Subsequently, the theta burst stimulation (TBS) protocol (30 trains of 6 pulses at 100 Hz) was applied to induce LTP. Then, the fEPSPs were recorded every 60 s for 90 min following TBS. The low-frequency stimulation (LFS) protocol (900 pulses of 1 Hz for 15 min) was applied to induce DEP. The fEPSPs were recorded every 60 s for 90 min after the LFS. The collected data were then analyzed using Clampfit 10.0 software (Molecular Devices, Sunnyvale, CA, USA). After the electrophysiological experiments, the mice were sacrificed, and their brains were utilized for further experiments, such as IHC, silver staining, or WB experiments.

### 4.12. Data Analysis

Statistical analyses were performed using GraphPad Prism 8 software. Differences between multiple groups were analyzed using one-way analysis of variance (ANOVA) with repeated measurements, which was conducted using the data in Figure 2, Figure 3, Figure 4, Figure 5 and Figure 7. Two-way ANOVA with repeated measurements was conducted on the data in Figure 6. Fisher’s LSD was used for pairwise comparisons. All data were presented as mean ± SEM. The significant differences were * *p* < 0.05, ** *p* < 0.01, *** *p* < 0.001, **** *p* < 0.0001, and ns, or not significant. Statistical significance was recognized as *p* < 0.05.

## 5. Conclusions

Transcranial magneto-acoustic stimulation (TMAS) and transcranial ultrasound stimulation (TUS) are both physical techniques for non-invasive brain stimulation. TMAS is an advantageous technique based on the use of a magnetic–acoustic coupling electric field to regulate the neuroelectric activity of deep brain regions. Our results showed that the effects of Aβ plaque clearance and the anti-cognitive impairment effects induced with TMAS treatment were significantly greater than those induced with TUS and sham treatments, and the improved performance of TMAS was mediated through microglial functions via the PI3K-AKT signaling pathway. This research suggests that TMAS treatment represents a favorable protective factor for AD pathological remission, and TMAS combined with other microglial regulators should therefore be explored for AD therapy.

## Data Availability

The authors confirm that the data supporting the findings of this study are available within the article.

## Supplementary Materials

The following supporting information can be downloaded at: https://www.mdpi.com/article/10.3390/ijms25094651/s1.

## References

[1] Gabrielli M., Prada I., Joshi P., Falcicchia C., D’Arrigo G., Rutigliano G., Battocchio E., Zenatelli R., Tozzi F., Radeghieri A. (2022). Microglial large extracellular vesicles propagate early synaptic dysfunction in Alzheimer’s disease. Brain.

[2] Passeri E., Elkhoury K., Morsink M., Broersen K., Linder M., Tamayol A., Malaplate C., Yen F.T., Arab-Tehrany E. (2022). Alzheimer’s Disease: Treatment Strategies and Their Limitations. Int. J. Mol. Sci..

[3] Karran E., Mercken M., De Strooper B. (2011). The amyloid cascade hypothesis for Alzheimer’s disease: An appraisal for the development of therapeutics. Nat. Rev. Drug Discov..

[4] Yoon S.S., Jo S.A. (2012). Mechanisms of Amyloid-beta Peptide Clearance: Potential Therapeutic Targets for Alzheimer’s Disease. Biomol. Ther..

[5] Gandy S., Heppner F.L. (2013). Microglia as dynamic and essential components of the amyloid hypothesis. Neuron.

[6] Yin Z., Herron S., Silveira S., Kleemann K., Gauthier C., Mallah D., Cheng Y., Margeta M.A., Pitts K.M., Barry J.L. (2023). Identification of a protective microglial state mediated by miR-155 and interferon-gamma signaling in a mouse model of Alzheimer’s disease. Nat. Neurosci..

[7] Clayton K., Delpech J.C., Herron S., Iwahara N., Ericsson M., Saito T., Saido T.C., Ikezu S., Ikezu T. (2021). Plaque associated microglia hyper-secrete extracellular vesicles and accelerate tau propagation in a humanized APP mouse model. Mol. Neurodegener..

[8] Bobola M.S., Chen L., Ezeokeke C.K., Olmstead T.A., Nguyen C., Sahota A., Williams R.G., Mourad P.D. (2020). Transcranial focused ultrasound, pulsed at 40 Hz, activates microglia acutely and reduces Abeta load chronically, as demonstrated in vivo. Brain Stimul..

[9] Park M., Hoang G.M., Nguyen T., Lee E., Jung H.J., Choe Y., Lee M.H., Hwang J.Y., Kim J.G., Kim T. (2021). Effects of transcranial ultrasound stimulation pulsed at 40 Hz on Abeta plaques and brain rhythms in 5xFAD mice. Transl. Neurodegener..

[10] Zhou X., Liu S., Wang Y., Yin T., Yang Z., Liu Z. (2019). High-Resolution Transcranial Electrical Simulation for Living Mice Based on Magneto-Acoustic Effect. Front. Neurosci..

[11] Wang H., Zhou X., Cui D., Liu R., Tan R., Wang X., Liu Z., Yin T. (2019). Comparative Study of Transcranial Magneto-Acoustic Stimulation and Transcranial Ultrasound Stimulation of Motor Cortex. Front. Behav. Neurosci..

[12] Wang Y., Feng L., Liu S., Zhou X., Yin T., Liu Z., Yang Z. (2019). Transcranial Magneto-Acoustic Stimulation Improves Neuroplasticity in Hippocampus of Parkinson’s Disease Model Mice. Neurotherapeutics.

[13] Lam S., Herard A.S., Boluda S., Petit F., Eddarkaoui S., Cambon K., Picq J.L., Buee L., Duyckaerts C., Haik S. (2022). Pathological changes induced by Alzheimer’s brain inoculation in amyloid-beta plaque-bearing mice. Acta Neuropathol. Com..

[14] Horwood J.M., Dufour F., Laroche S., Davis S. (2006). Signalling mechanisms mediated by the phosphoinositide 3-kinase/Akt cascade in synaptic plasticity and memory in the rat. Eur. J. Neurosci..

[15] Razani E., Pourbagheri-Sigaroodi A., Safaroghli-Azar A., Zoghi A., Shanaki-Bavarsad M., Bashash D. (2021). The PI3K/Akt signaling axis in Alzheimer’s disease: A valuable target to stimulate or suppress?. Cell Stress Chaperones.

[16] Liu Y., Liu F., Grundke-Iqbal I., Iqbal K., Gong C.X. (2011). Deficient brain insulin signalling pathway in Alzheimer’s disease and diabetes. J. Pathol..

[17] Steen E., Terry B.M., Rivera E.J., Cannon J.L., Neely T.R., Tavares R., Xu X.J., Wands J.R., de la Monte S.M. (2005). Impaired insulin and insulin-like growth factor expression and signaling mechanisms in Alzheimer’s disease—Is this type 3 diabetes?. J. Alzheimers Dis..

[18] Li N., Xiao K., Mi X., Li N., Guo L., Wang X., Sun Y., Li G.D., Zhou Y. (2022). Ghrelin signaling in dCA1 suppresses neuronal excitability and impairs memory acquisition via PI3K/Akt/GSK-3beta cascades. Neuropharmacology.

[19] Gabbouj S., Ryhanen S., Marttinen M., Wittrahm R., Takalo M., Kemppainen S., Martiskainen H., Tanila H., Haapasalo A., Hiltunen M. (2019). Altered Insulin Signaling in Alzheimer’s Disease Brain—Special Emphasis on PI3K-Akt Pathway. Front. Neurosci..

[20] Jung S.Y., Kim D.Y. (2017). Treadmill exercise improves motor and memory functions in cerebral palsy rats through activation of PI3K-Akt pathway. J. Exerc. Rehabil..

[21] Weinhard L., di Bartolomei G., Bolasco G., Machado P., Schieber N.L., Neniskyte U., Exiga M., Vadisiute A., Raggioli A., Schertel A. (2018). Microglia remodel synapses by presynaptic trogocytosis and spine head filopodia induction. Nat. Commun..

[22] Huang Y., Happonen K.E., Burrola P.G., O’Connor C., Hah N., Huang L., Nimmerjahn A., Lemke G. (2021). Microglia use TAM receptors to detect and engulf amyloid beta plaques. Nat. Immunol..

[23] Qiu Y., Shen X., Ravid O., Atrakchi D., Rand D., Wight A.E., Kim H.J., Liraz-Zaltsman S., Cooper I., Schnaider B.M. (2023). Definition of the contribution of an Osteopontin-producing CD11c(+) microglial subset to Alzheimer’s disease. Proc. Natl. Acad. Sci. USA.

[24] Wlodarczyk A., Lobner M., Cedile O., Owens T. (2014). Comparison of microglia and infiltrating CD11c^+^ cells as antigen presenting cells for T cell proliferation and cytokine response. J. Neuroinflamm..

[25] Blasi E., Barluzzi R., Bocchini V., Mazzolla R., Bistoni F. (1990). Immortalization of murine microglial cells by a v-*raf*/v-*myc* carrying retrovirus. J. Neuroimmunol..

[26] Sheppard O., Coleman M. (2020). Alzheimer’s Disease: Etiology, Neuropathology and Pathogenesis.

[27] Jo K.W., Lee D., Cha D.G., Oh E., Choi Y.H., Kim S., Park E.S., Kim J.K., Kim K.T. (2022). Gossypetin ameliorates 5xFAD spatial learning and memory through enhanced phagocytosis against Abeta. Alzheimers Res. Ther..

[28] Fang E.F., Hou Y., Palikaras K., Adriaanse B.A., Kerr J.S., Yang B., Lautrup S., Hasan-Olive M.M., Caponio D., Dan X. (2019). Mitophagy inhibits amyloid-beta and tau pathology and reverses cognitive deficits in models of Alzheimer’s disease. Nat. Neurosci..

[29] Jantti H., Sitnikova V., Ishchenko Y., Shakirzyanova A., Giudice L., Ugidos I.F., Gomez-Budia M., Korvenlaita N., Ohtonen S., Belaya I. (2022). Microglial amyloid beta clearance is driven by PIEZO1 channels. J. Neuroinflamm..

[30] Maccioni R.B., Munoz J.P., Barbeito L. (2001). The molecular bases of Alzheimer’s disease and other neurodegenerative disorders. Arch. Med. Res..

[31] Sharoar M.G., Palko S., Ge Y., Saido T.C., Yan R. (2021). Accumulation of saposin in dystrophic neurites is linked to impaired lysosomal functions in Alzheimer’s disease brains. Mol. Neurodegener..

[32] Di Meco A., Kemal S., Popovic J., Chandra S., Sadleir K., Vassar R. (2022). Poloxamer-188 Exacerbates Brain Amyloidosis, Presynaptic Dystrophies, and Pathogenic Microglial Activation in 5XFAD Mice. Curr. Alzheimer Res..

[33] Niu X., Yu K., He B. (2022). Transcranial focused ultrasound induces sustained synaptic plasticity in rat hippocampus. Brain Stimul..

[34] Kim H.J., Phan T.T., Lee K., Kim J.S., Lee S.Y., Lee J.M., Do J., Lee D., Kim S.P., Lee K.P. (2024). Long-lasting forms of plasticity through patterned ultrasound-induced brainwave entrainment. Sci. Adv..

[35] Huang X., Lin Z., Wang K., Liu X., Zhou W., Meng L., Huang J., Yuan K., Niu L., Zheng H. (2019). Transcranial Low-Intensity Pulsed Ultrasound Modulates Structural and Functional Synaptic Plasticity in Rat Hippocampus. IEEE Trans. Ultrason. Ferroelectr. Freq. Control.

[36] Ramachandran S., Niu X., Yu K., He B. (2022). Transcranial ultrasound neuromodulation induces neuronal correlation change in the rat somatosensory cortex. J. Neural Eng..

[37] Kong C., Ahn J.W., Kim S., Park J.Y., Na Y.C., Chang J.W., Chung S., Chang W.S. (2023). Long-lasting restoration of memory function and hippocampal synaptic plasticity by focused ultrasound in Alzheimer’s disease. Brain Stimul..

[38] Leinenga G., Gotz J. (2015). Scanning ultrasound removes amyloid-beta and restores memory in an Alzheimer’s disease mouse model. Sci. Transl. Med..

[39] Shin J., Kong C., Lee J., Choi B.Y., Sim J., Koh C.S., Park M., Na Y.C., Suh S.W., Chang W.S. (2019). Focused ultrasound-induced blood-brain barrier opening improves adult hippocampal neurogenesis and cognitive function in a cholinergic degeneration dementia rat model. Alzheimers Res. Ther..

[40] Lee C.H., Chen H.M., Yeh L.K., Hong M.Y., Huang G.S. (2012). Dosage-dependent induction of behavioral decline in *Caenorhabditis elegans* by long-term treatment of static magnetic fields. J. Radiat. Res..

[41] Hung Y.C., Lee J.H., Chen H.M., Huang G.S. (2010). Effects of static magnetic fields on the development and aging of *Caenorhabditis elegans*. J. Exp. Biol..

[42] Peng Y., Lu K., Li Z., Zhao Y., Wang Y., Hu B., Xu P., Shi X., Zhou B., Pennington M. (2014). Blockade of Kv1.3 channels ameliorates radiation-induced brain injury. Neuro-Oncology.

[43] El-Sherbeeny N.A., Ibrahiem A.T., Ali H.S., Farag N.E., Toraih E.A., Zaitone S.A. (2020). Carbamazepine conquers spinal GAP43 deficiency and sciatic Nav1.5 upregulation in diabetic mice: Novel mechanisms in alleviating allodynia and hyperalgesia. Arch. Pharm. Res..

[44] Wu T., Wang X., Cheng J., Liang X., Li Y., Chen M., Kong L., Tang M. (2022). Nitrogen-doped graphene quantum dots induce ferroptosis through disrupting calcium homeostasis in microglia. Part. Fibre Toxicol..

[45] Eder C., Klee R., Heinemann U. (1998). Involvement of stretch-activated Cl- channels in ramification of murine microglia. J. Neurosci..

[46] Demir I.E., Tieftrunk E., Schorn S., Saricaoglu O.C., Pfitzinger P.L., Teller S., Wang K., Waldbaur C., Kurkowski M.U., Wormann S.M. (2016). Activated Schwann cells in pancreatic cancer are linked to analgesia via suppression of spinal astroglia and microglia. Gut.

[47] Hu J., Chen Q., Zhu H., Hou L., Liu W., Yang Q., Shen H., Chai G., Zhang B., Chen S. (2023). Microglial Piezo1 senses Abeta fibril stiffness to restrict Alzheimer’s disease. Neuron.

[48] Wang J., Zhao M., Jia P., Liu F.F., Chen K., Meng F.Y., Hong J.H., Zhang T., Jin X.H., Shi J. (2020). The analgesic action of larixyl acetate, a potent TRPC6 inhibitor, in rat neuropathic pain model induced by spared nerve injury. J. Neuroinflam..

[49] Wang W., Li Y., Ma F., Sheng X., Chen K., Zhuo R., Wang C., Zheng H., Zhang Y.W., Bu G. (2023). Microglial repopulation reverses cognitive and synaptic deficits in an Alzheimer’s disease model by restoring BDNF signaling. Brain Behav. Immun..

[50] Kohman R.A., DeYoung E.K., Bhattacharya T.K., Peterson L.N., Rhodes J.S. (2012). Wheel running attenuates microglia proliferation and increases expression of a proneurogenic phenotype in the hippocampus of aged mice. Brain Behav. Immun..

[51] Nguyen P.T., Dorman L.C., Pan S., Vainchtein I.D., Han R.T., Nakao-Inoue H., Taloma S.E., Barron J.J., Molofsky A.B., Kheirbek M.A. (2020). Microglial Remodeling of the Extracellular Matrix Promotes Synapse Plasticity. Cell.

[52] Valentinova K., Tchenio A., Trusel M., Clerke J.A., Lalive A.L., Tzanoulinou S., Matera A., Moutkine I., Maroteaux L., Paolicelli R.C. (2019). Morphine withdrawal recruits lateral habenula cytokine signaling to reduce synaptic excitation and sociability. Nat. Neurosci..

[53] Picard K., Corsi G., Decoeur F., Di Castro M.A., Bordeleau M., Persillet M., Laye S., Limatola C., Tremblay M.E., Nadjar A. (2023). Microglial homeostasis disruption modulates non-rapid eye movement sleep duration and neuronal activity in adult female mice. Brain Behav. Immun..

[54] Gmitrov J. (2020). Static Magnetic Field Versus Systemic Calcium Channel Blockade Effect on Microcirculation: Possible Mechanisms and Clinical Implementation. Bioelectromagnetics.

[55] Li Q., Liao Z., Gu L., Zhang L., Zhang L., Tian X., Li J., Fang Z., Zhang X. (2020). Moderate Intensity Static Magnetic Fields Prevent Thrombus Formation in Rats and Mice. Bioelectromagnetics.

[56] Gmitrov J. (2007). Geomagnetic field modulates artificial static magnetic field effect on arterial baroreflex and on microcirculation. Int. J. Biometeorol..

[57] Okano H., Masuda H., Ohkubo C. (2005). Decreased plasma levels of nitric oxide metabolites, angiotensin II, and aldosterone in spontaneously hypertensive rats exposed to 5 mT static magnetic field. Bioelectromagnetics.

[58] Cheng L., Yang B., Du H., Zhou T., Li Y., Wu J., Cao Z., Xu A. (2022). Moderate intensity of static magnetic fields can alter the avoidance behavior and fat storage of *Caenorhabditis elegans* via serotonin. Environ. Sci. Pollut. R..

[59] Sinha A.S., Shibata S., Takamatsu Y., Akita T., Fukuda A., Mima T. (2024). Static Magnetic Field Stimulation Enhances Shunting Inhibition via a SLC26 Family Cl(-) Channel, Inducing Intrinsic Plasticity. J. Neurosci..

[60] Choi W., Cho H., Kim G., Youn I., Key J., Han S. (2022). Targeted thrombolysis by magnetoacoustic particles in photothrombotic stroke model. Biomater. Res..

[61] Kim T., Kim H.J., Choi W., Lee Y.M., Pyo J.H., Lee J., Kim J., Kim J., Kim J.H., Kim C. (2023). Deep brain stimulation by blood-brain-barrier-crossing piezoelectric nanoparticles generating current and nitric oxide under focused ultrasound. Nat. Biomed. Eng..

[62] Singer A., Dutta S., Lewis E., Chen Z., Chen J.C., Verma N., Avants B., Feldman A.K., O’Malley J., Beierlein M. (2020). Magnetoelectric Materials for Miniature, Wireless Neural Stimulation at Therapeutic Frequencies. Neuron.

[63] Wang Y., Xu Y., Dong S., Wang P., Chen W., Lu Z., Ye D., Pan B., Wu D., Vecitis C.D. (2021). Ultrasonic activation of inert poly(tetrafluoroethylene) enables piezocatalytic generation of reactive oxygen species. Nat. Commun..

[64] Sood A., Desseigne M., Dev A., Maurizi L., Kumar A., Millot N., Han S.S. (2023). A Comprehensive Review on Barium Titanate Nanoparticles as a Persuasive Piezoelectric Material for Biomedical Applications: Prospects and Challenges. Small.

[65] Zhu P., Chen Y., Shi J. (2020). Piezocatalytic Tumor Therapy by Ultrasound-Triggered and BaTiO(3) -Mediated Piezoelectricity. Adv. Mater..

[66] Chen S., Zhu P., Mao L., Wu W., Lin H., Xu D., Lu X., Shi J. (2023). Piezocatalytic Medicine: An Emerging Frontier using Piezoelectric Materials for Biomedical Applications. Adv. Mater..

[67] Ge M., Xu D., Chen Z., Wei C., Zhang Y., Yang C., Chen Y., Lin H., Shi J. (2021). Magnetostrictive-Piezoelectric-Triggered Nanocatalytic Tumor Therapy. Nano Lett..

[68] Di Ianni T., Morrison K.P., Yu B., Murphy K.R., de Lecea L., Airan R.D. (2023). High-throughput ultrasound neuromodulation in awake and freely behaving rats. Brain Stimul..

[69] He J., Zhu Y., Wu C., Wu J., Chen Y., Yuan M., Cheng Z., Zeng L., Ji X. (2023). Simultaneous multi-target ultrasound neuromodulation in freely-moving mice based on a single-element ultrasound transducer. J. Neural Eng..

[70] Song C., Knopfel T. (2016). Optogenetics enlightens neuroscience drug discovery. Nat. Rev. Drug Discov..

[71] Yuan Y., Yan J., Ma Z., Li X. (2016). Noninvasive Focused Ultrasound Stimulation Can Modulate Phase-Amplitude Coupling between Neuronal Oscillations in the Rat Hippocampus. Front. Neurosci..

[72] Yuan Y., Yan J., Ma Z., Li X. (2016). Effect of noninvasive focused ultrasound stimulation on gamma oscillations in rat hippocampus. Neuroreport.

[73] Nyborg W.L. (2001). Biological effects of ultrasound: Development of safety guidelines. Part II: General review. Ultrasound Med. Biol..

[74] Duck F.A. (2007). Medical and non-medical protection standards for ultrasound and infrasound. Prog. Biophys. Mol. Bio..

[75] Kim E.D., Won Y.H., Park S.H., Seo J.H., Kim D.S., Ko M.H., Kim G.W. (2019). Efficacy and Safety of a Stimulator Using Low-Intensity Pulsed Ultrasound Combined with Transcutaneous Electrical Nerve Stimulation in Patients with Painful Knee Osteoarthritis. Pain. Res. Manag..

[76] Rabut C., Yoo S., Hurt R.C., Jin Z., Li H., Guo H., Ling B., Shapiro M.G. (2020). Ultrasound Technologies for Imaging and Modulating Neural Activity. Neuron.

[77] Schafer M.E., Spivak N.M., Korb A.S., Bystritsky A. (2021). Design, Development, and Operation of a Low-Intensity Focused Ultrasound Pulsation (LIFUP) System for Clinical Use. IEEE Trans. Ultrason. Ferroelectr. Freq. Control.

[78] Ignell R., Hill S.R. (2022). The Golgi Method. Cold Spring Harb. Protoc..

[79] Lavenir I., Passarella D., Masuda-Suzukake M., Curry A., Holton J.L., Ghetti B., Goedert M. (2019). Silver staining (Campbell-Switzer) of neuronal alpha-synuclein assemblies induced by multiple system atrophy and Parkinson’s disease brain extracts in transgenic mice. Acta Neuropathol. Com..

[80] Li Y., Xu B., Ren X., Wang L., Xu Y., Zhao Y., Yang C., Yuan C., Li H., Tong X. (2022). Inhibition of CISD2 promotes ferroptosis through ferritinophagy-mediated ferritin turnover and regulation of p62-Keap1-NRF2 pathway. Cell Mol. Biol. Lett..

[81] Martinez G., Cappetta D., Telesca M., Urbanek K., Castaldo G., Dhellemmes M., Mele V.G., Chioccarelli T., Porreca V., Barbotin A.L. (2023). Cytochalasin D restores nuclear size acting on F-actin and IZUMO1 localization in low-quality spermatozoa. Int. J. Biol. Sci..

[82] Sun Y., Sommerville N.R., Liu J., Ngan M.P., Poon D., Ponomarev E.D., Lu Z., Kung J., Rudd J.A. (2020). Intra-gastrointestinal amyloid-beta1-42 oligomers perturb enteric function and induce Alzheimer’s disease pathology. J. Physiol..

[83] Kim J., Lee H.J., Park S.K., Park J.H., Jeong H.R., Lee S., Lee H., Seol E., Hoe H.S. (2021). Donepezil Regulates LPS and Abeta-Stimulated Neuroinflammation through MAPK/NLRP3 Inflammasome/STAT3 Signaling. Int. J. Mol. Sci..

[84] Tonelli M., Catto M., Sabate R., Francesconi V., Laurini E., Pricl S., Pisani L., Miniero D.V., Liuzzi G.M., Gatta E. (2023). Thioxanthenone-based derivatives as multitarget therapeutic leads for Alzheimer’s disease. Eur. J. Med. Chem..

[85] Jiang Y., Li K., Li X., Xu L., Yang Z. (2021). Sodium butyrate ameliorates the impairment of synaptic plasticity by inhibiting the neuroinflammation in 5XFAD mice. Chem.-Biol. Interact..

[86] Chu F., Li K., Li X., Xu L., Huang J., Yang Z. (2021). Graphene Oxide Ameliorates the Cognitive Impairment through Inhibiting PI3K/Akt/mTOR Pathway to Induce Autophagy in AD Mouse Model. Neurochem. Res..

