# Supplementary material for: Transcranial Magneto-Acoustic Stimulation Protects Synaptic Rehabilitation from Amyloid-Beta Plaques via Regulation of Microglial Functions
Source: Int J Mol Sci. 2024 Apr 24;25(9):4651. doi: 10.3390/ijms25094651 (PMC11083601; doi:10.3390/ijms25094651)
Supplement: Supplementary file 1 [file ijms-25-04651-s001.zip › ijms-2920084-supplementary.pdf]

1.1. The specification of the transducer and the distribution of the ultrasound pressure field.

A

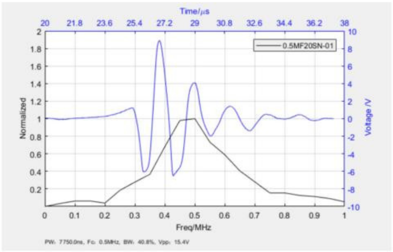

B

Performance index	Measured value
Center frequency (kHz)	500
-6dB bandwidth (%)	40.8
Echo peak（V）	15.4

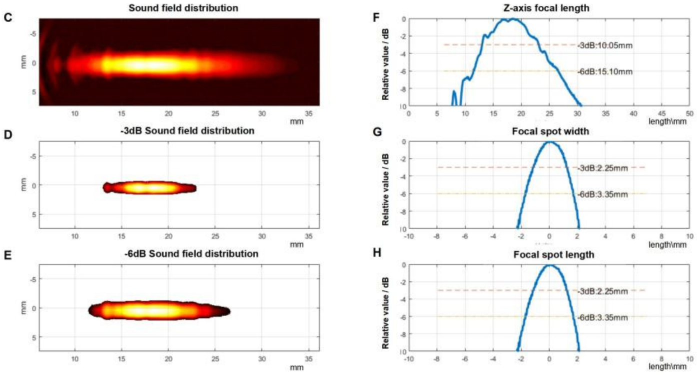

I

Performance index	Measured value
Focal length (mm)	18.6
-3dB Focal column length (mm)	10.05
-3dB Focal column width (mm)	2.25
-6dB Focal column length (mm)	15.10
-6dB Focal column width (mm)	3.35

Figure S1. Measurement results. (A) The specification of the transducer. (B) Measured values for the the transducer. (C) Sound field distribution. (D) -3dB Sound field distribution. (E) -6dB Sound field distribution. (F) Z-axis focal length. (G) Focal spot width. (H) Focal spot length. (I) Measured values for the distribution of the ultrasound pressure field.

1.2.1 The model mouse of microglia exhaustion is established using PLX3397

Our current findings suggest that TUS/TMAS treatment effectively modulates Aβ phagocytic microglia. To further investigate whether microglia mediate the protective effects of TUS/TMAS treatment against amyloid deposition, we employed the CSF1R inhibitor PLX3397 at 290 mg/kg chow to deplete the microglia in 5xFAD mice. After a period consuming the diet with PLX3397, we observed a significant reduction in microglial numbers, as confirmed by Iba1 staining (-PLX3397 vs. +PLX3397; p = 0.0022; Figure S2A,B) and in the WB experiments (-PLX3397 vs. +PLX3397; p = 0.0351; Figure S2C,D). To further evaluate the safety of the PLX3397 treatment, we conducted HE staining to examine major organs such as the heart, liver, spleen, lungs, and kidneys. The results revealed no evident damage or abnormalities in these organs (Figure S2E), indicating that the PLX3397 treatment did not have a detrimental effect on the major organs of 5xFAD mice.

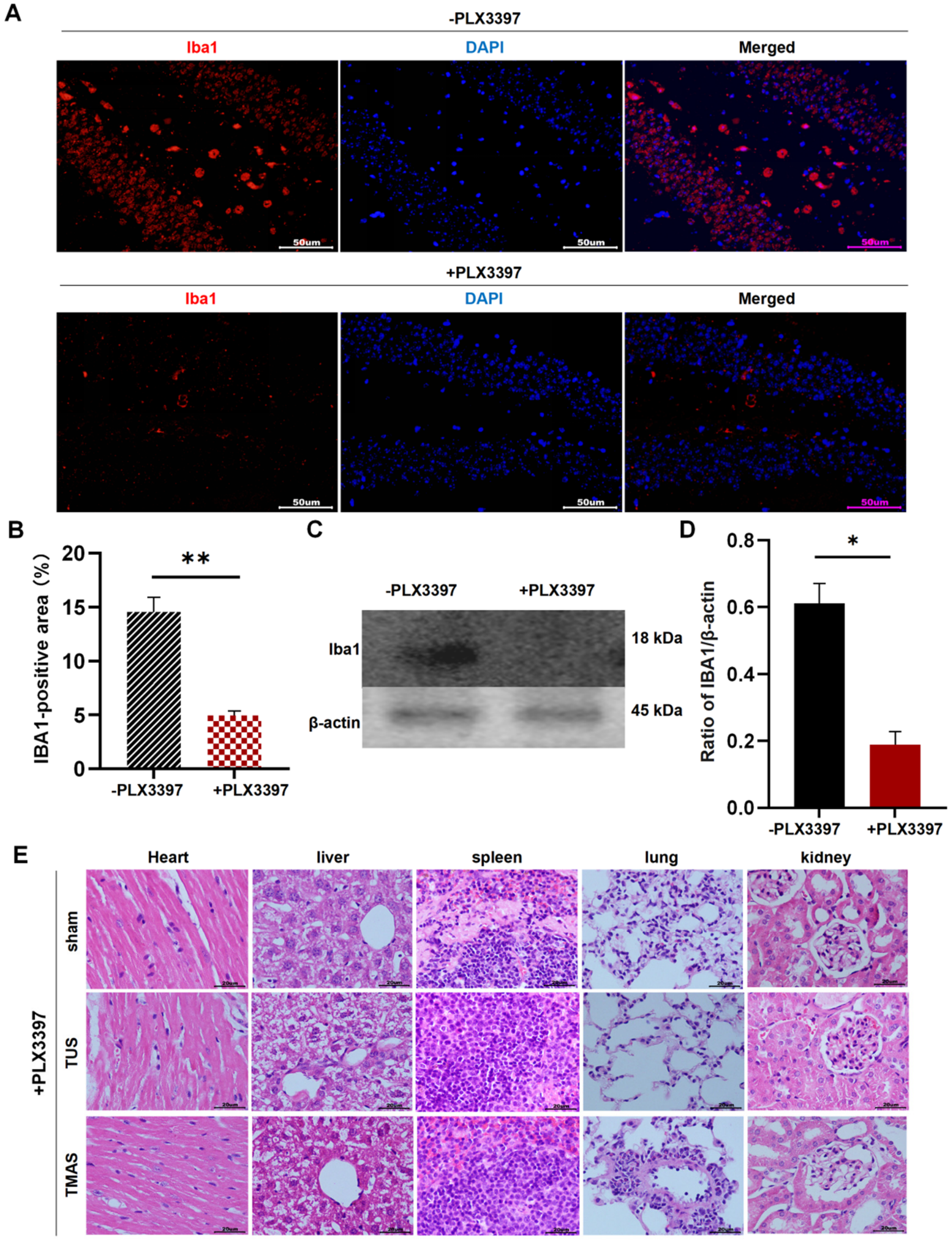

Figure S2. The microglial deficiency model was successfully established in 5xFAD mice after oral administration of PLX3397. (A,B) Coronal sections were stained with Iba1 (red) for microglia and DAPI (blue) for nuclei (n = 3 mice, 6 fields of each group for analysis). Original magnification, ×200; scale bar, 50 μm. (C,D) WB experiments showed that the number of microglia in the brains of 5xFAD mice was significantly decreased after oral administration of PLX3397 (n = 3 mice for each group). (E) HE staining confirmed that the heart, liver, spleen, lungs, and kidneys of mice were not significantly affected with PXL3397 treatment. Original magnification, ×400; scale bar, 20 μm. All data in the results are expressed as mean ± SEM. * $p < 0.05$; ** $p < 0.01$.

1.2.2 Data Analysis

Paired Student's t-test was used to analyze Figure 2. All data were presented as mean ± SEM. The significant differences were * $p < 0.05$, ** $p < 0.01$, *** $p < 0.001$, and **** $p < 0.0001$, and ns, not significant. Statistical significance was recognized as $p < 0.05$.
